# Bacterial cellulose: a versatile biopolymer for wound dressing applications

**DOI:** 10.1111/1751-7915.13392

**Published:** 2019-03-05

**Authors:** Raquel Portela, Catarina R. Leal, Pedro L. Almeida, Rita G. Sobral

**Affiliations:** ^1^ Laboratory of Molecular Microbiology of Bacterial Pathogens UCIBIO@REQUIMTE Departamento de Ciências da Vida Faculdade de Ciências e Tecnologia Universidade Nova de Lisboa 2829‐516 Caparica Portugal; ^2^ Área Departamental de Física ISEL ‐ Instituto Superior de Engenharia de Lisboa Instituto Politécnico de Lisboa Rua Conselheiro Emídio Navarro 1 P‐1959‐007 Lisboa Portugal; ^3^ CENIMAT/I3N Departamento de Ciência dos Materiais Faculdade Ciências e Tecnologia Universidade Nova de Lisboa 2829‐516 Caparica Portugal

## Abstract

Although several therapeutic approaches are available for wound and burn treatment and much progress has been made in this area, room for improvement still exists, driven by the urgent need of better strategies to accelerate wound healing and recovery, mostly for cases of severe burned patients. Bacterial cellulose (BC) is a biopolymer produced by bacteria with several advantages over vegetal cellulose, such as purity, high porosity, permeability to liquid and gases, elevated water uptake capacity and mechanical robustness. Besides its biocompatibility, BC can be modified in order to acquire antibacterial response and possible local drug delivery features. Due to its intrinsic versatility, BC is the perfect example of a biotechnological response to a clinical problem. In this review, we assess the BC main features and emphasis is given to a specific biomedical application: wound dressings. The production process and the physical–chemical properties that entitle this material to be used as wound dressing namely for burn healing are highlighted. An overview of the most common BC composites and their enhanced properties, in particular physical and biological, is provided, including the different production processes. A particular focus is given to the biochemistry and genetic manipulation of BC. A summary of the current marketed BC‐based wound dressing products is presented, and finally, future perspectives for the usage of BC as wound dressing are foreseen.

## Introduction

Cellulose is the most abundant naturally occurring polymer obtained from renewable sources. It consists of a linear homopolysaccharide composed by β‐d‐glucopyranose units linked by β‐1,4 glycosidic bonds (Cannon and Anderson, 1991). The most commercially exploited natural source of cellulose is wood, due to its high availability that meets the demands of the paper industry (Klemm *et al*., 2005). However, a variety of plants also contains large amounts of cellulose, such as hemp, flax or cotton (Fernandes *et al*., 2017). In addition to these sources, cellulose can be produced, among others, by seaweed, fungi and some species of bacteria, being the most noteworthy the non‐pathogenic bacteria of the genus *Komagateibacter*, such as *K. xylinus*, former Acetobacter and Gluconacetobacter (Brown, 1886). Several strains of *K. xylinus* produce extracellular cellulose forming a biofilm of varying thickness with the purpose of maintaining a high oxygenation of the colonies near the surface, which serves as a protective barrier against drying, natural enemies and radiation.

Besides being biodegradable, non‐toxic and biocompatible, one of the major advantages of bacterial cellulose (BC) over vegetal cellulose is its unique native purity that allows for its direct utilization. It is chemically equivalent to plant cellulose, but it is free of by‐products such as lignin, pectin, hemicellulose and other constituents of lignocellulosic materials (Klemm *et al*., 2001; Rahman and Netravali, 2016). It is obtained by fermentation and only contains microbial cells, nutrients and other secondary metabolites that can be easily removed, yielding highly pure cellulose. Although BC molecular formula is similar to plant cellulose, BC mechanical and physical outstanding properties emerge from its unique 3D structure that differs significantly from that of vegetal source, as BC aggregates to form long fibrils of 1.5 nm width, providing higher surface area, elasticity, resistance and flexibility. BC presents a unique reticulate network of thin fibres with a diameter more than 100 times smaller than that of plant‐derived fibres (Klemm *et al*., 2005).

BC has many intrinsic characteristics that make it an ideal scaffold for protecting injured tissues through wound dressings, especially for burn wounds, tissue regeneration and as temporary skin substitutes. Some of advantageous features of BC for these particular applications are the fact that it is non‐toxic, non‐carcinogenic and biocompatible, and it has the capacity to retain moisture, absorb exudates from the injured tissue and accelerate granulation (Li *et al*., 2015; Khalid *et al*., 2017).

The largest organ in the human body is the skin. Three layers, the epidermis, dermis and the fat layer, also known as hypodermis, compose the skin. The epidermis is the external layer of the skin, having the critical function of maintaining homeostasis of the body internal environment and at the same time protecting the body from the external environment and from potential pathogenic bacteria. The dermis is where all the blood vessels, nerves, hair follicles, oil and sweat glands are located. In its native state, skin is dry and acidic in nature (pH between 4 and 6.8) being keratinocytes the skin cells responsible for producing skin lipids and to maintain hydration levels. Altered skin integrity may be due to systemic factors, such as the nutritional status of the individual, vascular disease, heart conditions and diabetes, among others, or to extrinsic episodes such as accidents, immobility, pressure and surgical procedures. When an individual suffers severe damage to large areas of skin, such as burns, he is exposed to decreased local function, dehydration and infections that can result in loss of limb and sometimes death. The wound healing process comprises a complex series of biological processes aiming to restore the skin barrier function (prevent dehydration and bacterial infection). However, skin regeneration and wound healing can be slow and lead to chronic inflammation, especially in burn patients with additional systemic impairments (Rowan *et al*., 2015).

In 2016, the American Burn Association reported 486 000 burn‐related injuries receiving medical treatment in the United States, including 40 000 hospitalizations (American Burn Association, 2016). Although the survival rate has considerably increased to 96.8%, severe burn injuries are difficult to manage, requiring extended hospital stays and expensive treatment choices (American Burn Association, 2016). The current standard of care for closing burn wounds and preventing wound sepsis includes early surgical excision of the damaged necrotic tissue followed by complete coverage of the exposed area (Rowan *et al*., 2015).

Another relevant skin condition for wound dressing application is cancer lesions, such as basal‐cell carcinoma or skin lesions related to chemotherapy and radiation therapy. Larger lesions are usually vascularized and necrotized, being responsible for high amounts of drainage.

For all these skin lesion conditions, the perfect wound dressing must maintain the moisture of the wound, allow for oxygen exchange, adsorb wound exudate, accelerate re‐epithelialization for wound closure, reduce pain and healing time, and prevent infection. However, also additional and specific treatments adapted to the needs of each individual lesion are needed. All these requirements demand for a creative, integrative and flexible dressing application.

In health‐related applications, natural‐derived polymers present several advantages when compared with synthetic ones, such as biocompatibility, biodegradability and/or biological activity, as most of them are present in the structural tissues of living organisms. Currently worldwide, burn wound and skin graft donor site treatments vary widely, and a large number of different wound dressing materials are available for their treatment (Voineskos *et al*., 2009). BC features, such as high porosity, high water retention capacity, high mechanical strength in the wet state, low density, biocompatibility, non‐toxicity and biodegradability, make BC an outstanding material that is suitable for technological applications, particularly in the fields of biomedicine and pharmacology (Hu *et al*., 2014).

BC has already been used quite successfully in wound healing applications, proving that it could become a high‐value product in the field of biotechnology (Klemm *et al*., 2001; Czaja *et al*., 2006). In fact, biomedical devices have gained a significant amount of attention because of an increased interest in tissue‐engineered products for both wound care and the regeneration of damaged or diseased organs (Czaja *et al*., 2007). The use of BC as a wound dressing scaffold material saw light during the early 1980s (Fontana *et al*., 1990) and has been constantly improved.

Cellulose biosynthesis in bacteria is a multistep process involving individual genes and an operon called *bcs*ABCD (bacterial cellulose synthesis), first identified in *K. xylinus* (Umeda *et al*., 1999), encoding proteins and enzymes that associate the linear polymerization of glucose with the formation of a 3D structure of cellulose (Ross *et al*., 1991). Although *bcsA* and *bcsB* are essential genes, the maximum yield of BC production is only achieved with the expression of the entire operon, in which composition and structural organization are highly diverse among species (Karnezis *et al*., 2003; Perez‐Mendoza *et al*., 2015).

**Figure 1:**
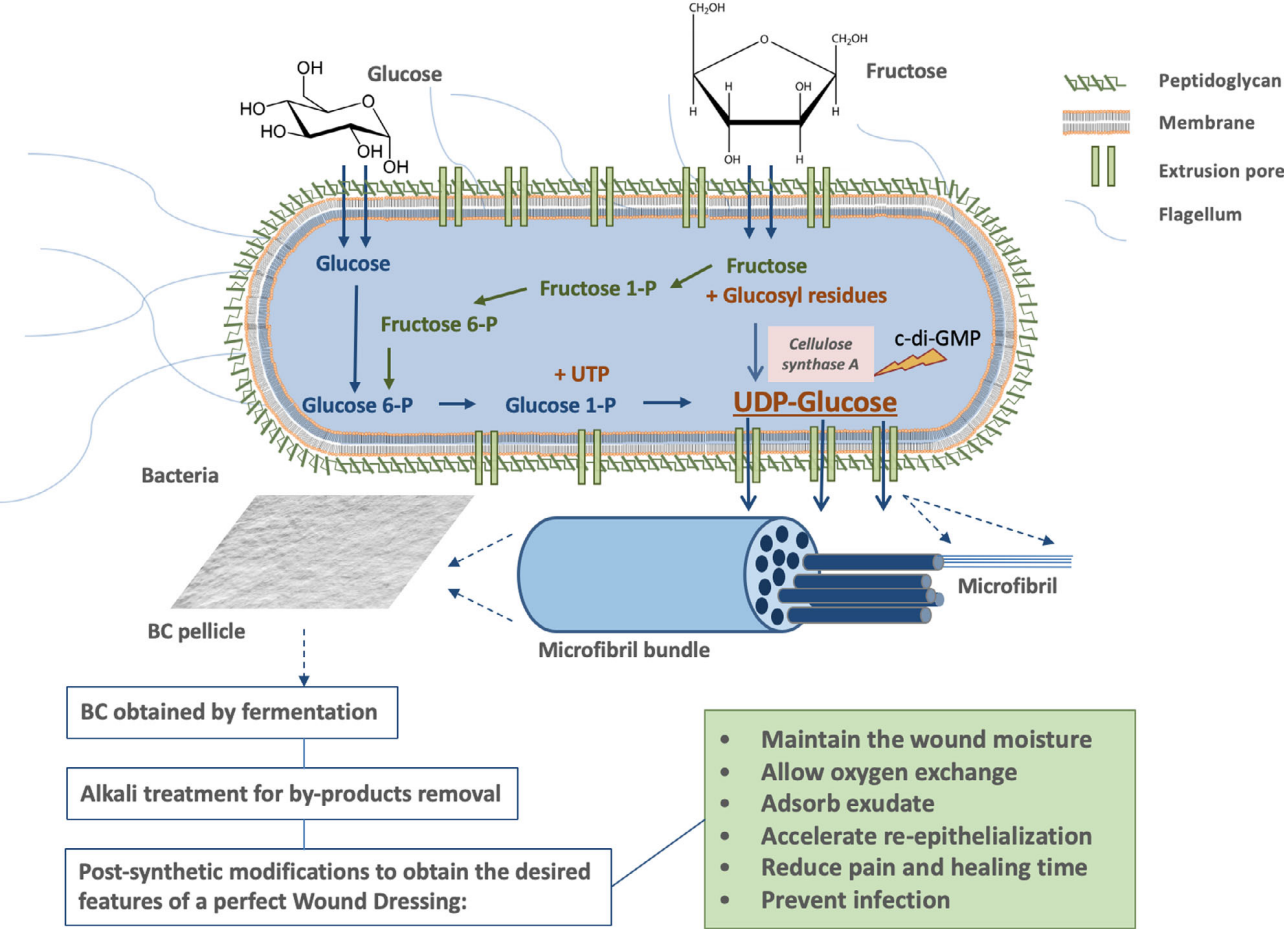
Schematic depiction of the steps involved in the production of a BC‐based wound dressing, from the molecular mechanism of UDP‐glucose biosynthesis in bacteria to the BC post‐synthetic modifications performed, highlighting the three‐dimensional structure formed by the secreted chains of glucose and the features desired to be present in a wound dressing‐based material.

The biosynthetic pathway of the cellulose exopolysaccharide begins with the isomerization of a glucose 6‐phosphate molecule into glucose 1‐phosphate. This intermediary reacts with UTP, forming uridine‐5′‐diphosphate‐alfa‐D‐glucose (UDP‐glucose), that is polymerized into linear ‐ 1,4 glucan chains in a reaction catalysed by cellulose synthase A that is activated by cyclic‐di‐GMP, into linear ‐ 1,4 glucan chains. The newly formed cellulose chains are then secreted across the cell wall through 50–80 extrusion pores, aligned along the cell long axis as illustrated in Fig. 1 (Kimura *et al*., 2001; Krasteva *et al*., 2017).

**Figure 2:**
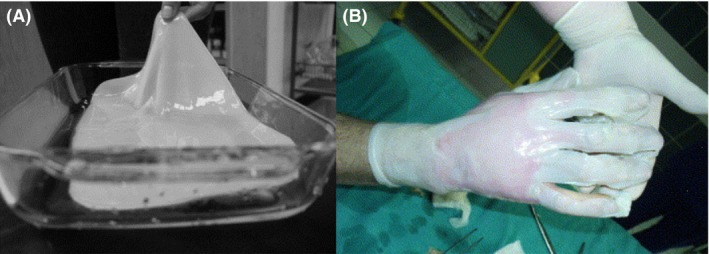
Bacterial cellulose. A. Hydrated BC membrane with high mechanical strength. B. BC wound dressing applied on a wounded hand. The BC's physical properties permits an excellent moulding to the wounded area (image courtesy of Center of Burn Healing, Siemianowice Slaskie, Poland), Reprinted from Biomaterials, Vol 27 (2), W. Czaja, A. Krystynowicz, S. Bielecki, R.M. Brown Jr., Microbial cellulose — the natural power to heal wounds, Pages No. 145–151, Copyright (2006), with permission from Elsevier.

After completing this first degree of organization – polymer formation – the linear chains are assembled into nanofibres of 10–15 polymer chains and are subsequently arranged into microfibrils (100 times smaller than those of plant cellulose) and then into microfibril bundles. The grouping of such bundles results in cellulose ribbons of 3–4 nm thickness and 70–80 wideness (Fig. 1), creating a 3D network that is stabilized through hydrogen bonds that establish intra‐ and interchemical links between the sheets of cellulose, forming a thick and gelatinous membrane characterized by a high mechanical strength (Fig. 2A) combined with a high malleability that allows it to perfectly mould to the wounded area (Fig. 2B) (Chawla *et al*., 2009).

## Intrinsic features of bacterial cellulose

### Mechanical properties of bacterial cellulose

The structural arrangement of the BC fibres confers mechanical properties that differentiate BC from plant‐derived cellulose, including a higher degree of crystallinity (84–89%) (Czaja *et al*., 2004) and absence of other contaminating polymers, allowing for a simple process of purification (Sani and Dahman, 2009). Also, the numerous hydroxyl groups that are available in glucose allow to establish interactions with over 90% of water molecules, which results in a high capacity of water retention (Gelin *et al*., 2007).

Despite the natural features of the BC polymer, the physical properties of BC strongly depend on the manufacturing and processing conditions. For instance, for dried samples of BC, it was found that typically, BC presents a tensile strength of around 240 MPa, a Young modulus of around 10 GPa and a maximum strain in the order of 3%, although it is estimated that the modulus of a single filament of BC can be as high as 114 GPa (Fernandes *et al*., 2013). The presented values show some dispersion between studies, for example, it is obtained for dried samples of BC a tensile strength of 400 MPa and an elongation at break of 10 % (Klemm *et al*., 2005). On hydrated BC samples (98% water content) of dry‐fabricated BC biofilm, the mechanical properties were in the order of: tensile strength of 380 kPa, maximum strain of 21% and a water vapour transmission rate of 2900 g m^−2^ day^−1^ (Fernandes *et al*., 2017). Also, for these samples the compression modulus was determined and found to be approximately 0.06 MPa (Klemm *et al*., 2001). Regarding the morphological properties on hydrated samples, it was found that BC had a specific surface area of approximately 60 m^2^ g^−1^, a specific pore volume of around 0.2 cm^3^ g^−1^ and an average pore diameter of around 13 nm (Qiao *et al*., 2015).

The above‐described characteristics of BC, together with the biocompatibility, non‐toxicity, cost‐effectiveness, formability, softness and the fact that the synthesis of BC can be highly adjusted for the optimization of these features and to incorporate secondary components, such as antibiotics into its pores, prove the existence of the fundamental properties ideal for wound dressing applications. In this sense, rheological characterization appears as a valuable tool to the design and optimization of BC materials, allowing to determine their response when subjected to a shear stress (Rebelo *et al*., 2018) and access, for instance, the time‐dependant rheological behaviour in such systems (Gao *et al*., 2016; Basu *et al*., 2017) or the elastic character of BC in function of its water content (Rebelo *et al*., 2018).

### Bacterial cellulose water holding/release capacity

Some of the main features required for wound dressings, in particular for burn treatment, are the water content and water‐retaining properties, in order to maintain the wound hydrated, as well as to be able to absorb large amounts of exudates.

**Figure 3:**
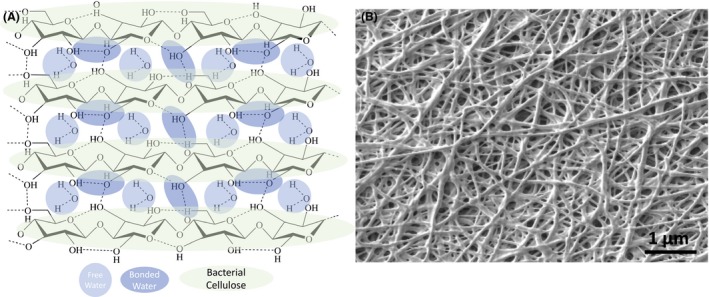
Bacterial cellulose. A. Molecular structure of hydrated BC. B. Typical microscopic BC fibre film morphology.

BC has a complex molecular structure, with water molecules bonded through hydrogen bonds. The BC fibres are composed of linear chains of glucan units linked through β‐1,4 glycosidic bonds. The glucan chains are linked through inter‐ and intramolecular hydrogen bonds, allowing BC to be mechanically robust while maintaining elasticity. The free water (unbonded) that is able to penetrate and to exit the BC molecular structure is responsible for the maintenance of the hydration level that is crucial for the wound dressing application (Fig. 3A).

Proper moisture control usually increases the healing rates, shields the wound from infections, diminishes pain and reduces the global healthcare expenses (Agarwal *et al*., 2011). Furthermore, water absorption and holding capacities allow to charge liquid drugs and bioactive compounds on the wound dressing material (Shah *et al*., 2013). The capability to maintain humidity also avoids the dehydration of the wound dressing and so prevents it from attaching to the wound, thus defending the tissue from exposure and diminishing the pain throughout the dressing exchange (Ovington, 2007). A typical SEM image of a non‐woven BC fibre structure is presented in Fig. 3B.

The presence of exudates is known to cause separation of the tissue layers of the wound, which makes the healing process slower. Therefore, exudates should be eliminated from the wound (Hedlund, 2007) and good drainage capacity emerges as a decisive criterion in the application of dressings. However, it is necessary to ensure that the wound maintains the necessary hydration for the healing process to occur in the best manner, requiring the dressing to balance the absorption and release of liquid (Davidson, 2015). With this aim, the properties of the dressing with respect to the ability to retain water and the ability to release water have been the subject of several studies to characterize new BC systems that can be applied to wound dressings. The water holding capacity (WHC) and water release rate (WRR) values appear as quantitative physical parameters for this evaluation, which are strongly dependent on the physicochemical and on the structural characteristics of the BC system. In particular, the available surface area and pore size distribution (Ul‐Islam *et al*., 2012), as well as the presence of hydrophilic additives in the BC system, are known to introduce significant changes in WHC and WRR values. Another quality index for wound dressings is the water vapour transmission rate (WVTR). An excessively high WVTR accelerates the dehydration and scabbing of a wound, whereas an excessively low WVTR causes wound fluids to accumulate, impedes healing and raises the risk of bacterial contamination. A desirable WVTR is 2500 –3000 g m^−2^ day^−1^ (Paul and Sharma, 2004; Li *et al*., 2014a).

BC water holding capacity ranges from 60 to 700 times of its dry weight, depending on the synthesis conditions. In typical statically cultured pellicles, BC represents approximately 1% of the total weight, with the rest being water (Yamanaka *et al*., 1989; Okiyama *et al*., 1992). A possible explanation for such high hydrophilicity level relates to the fact that the assembly of the cellulose ribbons occurs extracellularly, in the liquid medium, and numerous micelles are then formed trapping large amounts of liquid. Moreover, the hydrophilicity of the BC pellicle results in part from the wide internal surface area of the interstitial space of the wet pellicle. In fact, upon drying, BC exhibits poor rehydration due to high crystallinity, restricting its applications as a dressing material (Huang *et al*., 2010). In order to improve this point, different strategies have been implemented, altering the BC structure with the aim of increasing its water holding/release capacity.

### Bacterial cellulose structure – pore size and fibre morphology

The BC WHC relates directly with the available pore volume and surface area. A more compact BC structure, presenting denser fibril arrangements with reduced pore volume and surface area (Wang *et al*., 2012), is associated with a decrease in the WHC values. In such fibril structures, the available space and number of trapping sites to capture water molecules are diminished. On the other hand, a more compact BC structure is associated with lower WRR values. The denser microfibrils result in a greater amount of water retained in the system due to the formation of hydrogen bonds and a smaller amount of free bulk water (Gelin *et al*., 2007), which prevents water evaporation. Knowing these counteracting effects associated with the BC structure on the ability to hold and to release water, the possibility to access and adjust the BC structural parameters during the biosynthesis process or by post‐synthetic modifications has been the focus of several studies during the last decade. Alteration of the fermentation settings, such as culture growth conditions, culture media components and the presence of specific additives in the BC synthetic process, causes changes in the structural properties, namely in the crystallinity and in the fibre morphology (Sulaeva *et al*., 2015).

Investigations on the dependence of BC water holding and retention ability on structural characteristics showed that a reduction in pore size and surface area, obtained through BC modification, resulted in a decrease in WHC and in an increase in WRR (Ul‐Islam *et al*., 2012). In this study, the BC structure was modified by the addition of a single sugar‐linked glucuronic acid‐based oligosaccharide (SSGO) in the culture media and also via post‐synthetic treatment with inorganic montmorillonite clay. This direct relationship between WHC and pore size was also demonstrated in other works. A more compact BC structure with reduced porosity was obtained via a synthetic procedure in the presence of hydroxypropyl methylcellulose (HPMC), showing a reduced WHC value (Huang *et al*., 2011). On the contrary, BC structures with enhanced porosity presented a greater WHC value. BC synthesized in the presence of carboxymethylcellulose (CMC) in the culture media revealed a network with broader ribbons, due to the adhesion of CMC into the surface of BC fibrils, with greater WHC (Chen *et al*., 2016). An increased pore size distribution was also found in a BC structure with loose fibril arrangement (Grande *et al*., 2009), which corresponded to a greater WHC value (Seifert *et al*., 2004; Yu *et al*., 2011).

Higher porosity and therefore increased water holding/release ability can also be reached by post‐synthetic modifications of BC. A highly porous BC structure capable to absorb at least seven times more water than pure BC resulted from the use of foaming agents (Yin *et al*., 2012). Paximada *et al*. observed that a short ultrasonic pre‐treatment (1 min) applied to BC aqueous suspensions induced fibrils’ breakdown, which resulted in the increase in WHC, along with the viscosity and the solid‐like character of the samples (Paximada *et al*., 2016).

Mechanical and electrochemical properties were studied for BC under different water contents (100%, 80% and 50%), for which a progressive stiffening and increasing resistance with lower capacitance were observed after partial dehydration. A theoretical model for predicting BC water loss was developed and applied, which allows an understanding of the structural changes presented by post‐dried BC (Rebelo *et al*., 2018).

### Bacterial cellulose structure – synthesis components

In general, pore size reduction can be obtained by the addition of a secondary component into the BC fibre network, causing pore filling. Yet, WHC and WRR can be influenced by the nature of the secondary component itself.

The presence of highly hydrophilic chitosan promoted the absorption of larger amounts of water in BC/chitosan composites, when compared with native BC, even though with a reduced porosity. WHC was increased due to the capability of chitosan molecules to establish hydrogen bonds at the same time with BC fibrils and with water molecules. Simultaneously, WRR was increased, caused by the reduced porosity. These results highlight the importance of additive choice when directing the characteristics of BC towards the desired dressing need (Ul‐Islam *et al*., 2012).

The progressively greater water absorption capacity of BC/chitosan films with increasing chitosan content was early described by Phisalaphong and Jatupaiboon (2008); Phisalaphong *et al*. (2008). The chitosan molar mass was also observed to influence WHC, where a greater WHC was associated with a higher molar weight. A more compact structure with smaller pore size was described for BC/chitosan composites by Lin *et al*. (2013). WHC and WRR presented no significant difference when comparing modified samples with pure BC, although the dressings obtained from modified BC provided a suitable moisture environment for wounds with low‐ and mid‐range amounts of exudate.

The influence of other highly hydrophilic compounds on WHC and WRR was also reported. The incorporation of alginate in the BC structure gave rise to a nanoporous structure and to a decrease in pore diameter, but an increase in water uptake ability was described (Phisalaphong *et al*., 2008). The increased WHC observed in such BC/alginate films was justified by Chiaoprakobkij *et al*., who associated the disruption of the hydrogen bonds between cellulose fibres to the mixture with the other component. The greater capability to water uptake observed in dried sponges was considered to better support exudate adsorption (Chiaoprakobkij *et al*., 2011).

*Aloe vera* gel also appeared as an advantageous component to be included in wound dressings. When introduced to the BC structure during the biosynthesis process at a gel content of less than 30%, *aloe vera* gel increased the WHC by about 1.5‐fold compared to the non‐modified material. Improvement of water vapour permeability was also observed in addition of BC/*aloe vera* gel (Saibuatong and Phisalaphong, 2010).

Recently, Chang and Chen (2016) characterized the physical properties of HOBC/chitosan/alginate films for wound dressing. The WVTR of BC films prepared using gel solutions of 98.0% and 98.5% water content was 2865 and 3034 g m^−2^ day^−1^, respectively, and was close to the ideal dressing. However, the lower fluidity of a gel solution with 98.0% water content was not favourable for moulding. The gel solution with 98.5% water content exhibited the most desirable mechanical properties, hydrophilicity and WVTR.

The composites BC/hydrophilic additive presented similar properties to the systems BC/chitosan, BC/alginate and BC/*aloe vera* gel, showing the possibility to access the adjustment of WCH and WRR during composite preparation and the production of materials with efficient dressing characteristics.

Other possible systems considered hydrophilic synthetic polymers to produce BC composites for wound dressing applications. For example, BC modified with glycerine, used as a plasticizer, was observed to promote an excellent skin moisturizing effect. Good biocompatibility and enhanced malleability suggested the use of this material in dry wound treatment, such as those appearing due to psoriasis and atopic dermatitis (Almeida *et al*., 2014).

The liquid absorption characteristics of BC may be considerably altered by adding hydrophilic synthetic polymers, prepared as anion exchange membranes, such as poly‐AEM. The BC/poly‐AEM composites presented increased swelling capacity, from 100% up to 6200%, in comparison with the non‐modified BC and with the composite material respectively. Such swelling performance resulted from the conjugation of the hydrophilic nature of the synthetic component together with its capacity to prevent the collapse of the BC structure during the drying process (Figueiredo *et al*., 2015).

BC/acrylic hydrogels also presented great improvement in the swelling ratio of up to 4000–6000%. These results were observed in *in vivo* experiments, in which the use of BC composites confirmed promotion of burn healing with enhanced epithelialization and fibroblast proliferation (Mohamad *et al*., 2014). Such BC composites were considered as a promising material for burn dressing.

## Bacterial cellulose composites

A composite is a material that combines at least two distinct materials, with a clear interface between them, acquiring complementary properties of its constituents. Typically, the aim of producing composite materials is to provide the base material with properties (from the reinforcement) that it did not possess by itself, and that are required for a specific application. In most cases, these properties could not be achieved with the isolated base component. The reinforcing phase, which may consist of fibres (Lazarini *et al*., 2016), particles (Galateanu *et al*., 2015), sheets (layers) (Foong *et al*., 2018), interpenetrating networks (Lin *et al*., 2014) or cells, is dispersed in the so‐called matrix or continuous phase (usually the one presenting a higher percentage). The properties of the composites depend on both the nature of the materials employed and the degree of bonding between them through the interface. All type of materials can be used to produce composites ranging from polymers to ceramics or metals.

**Figure 4:**
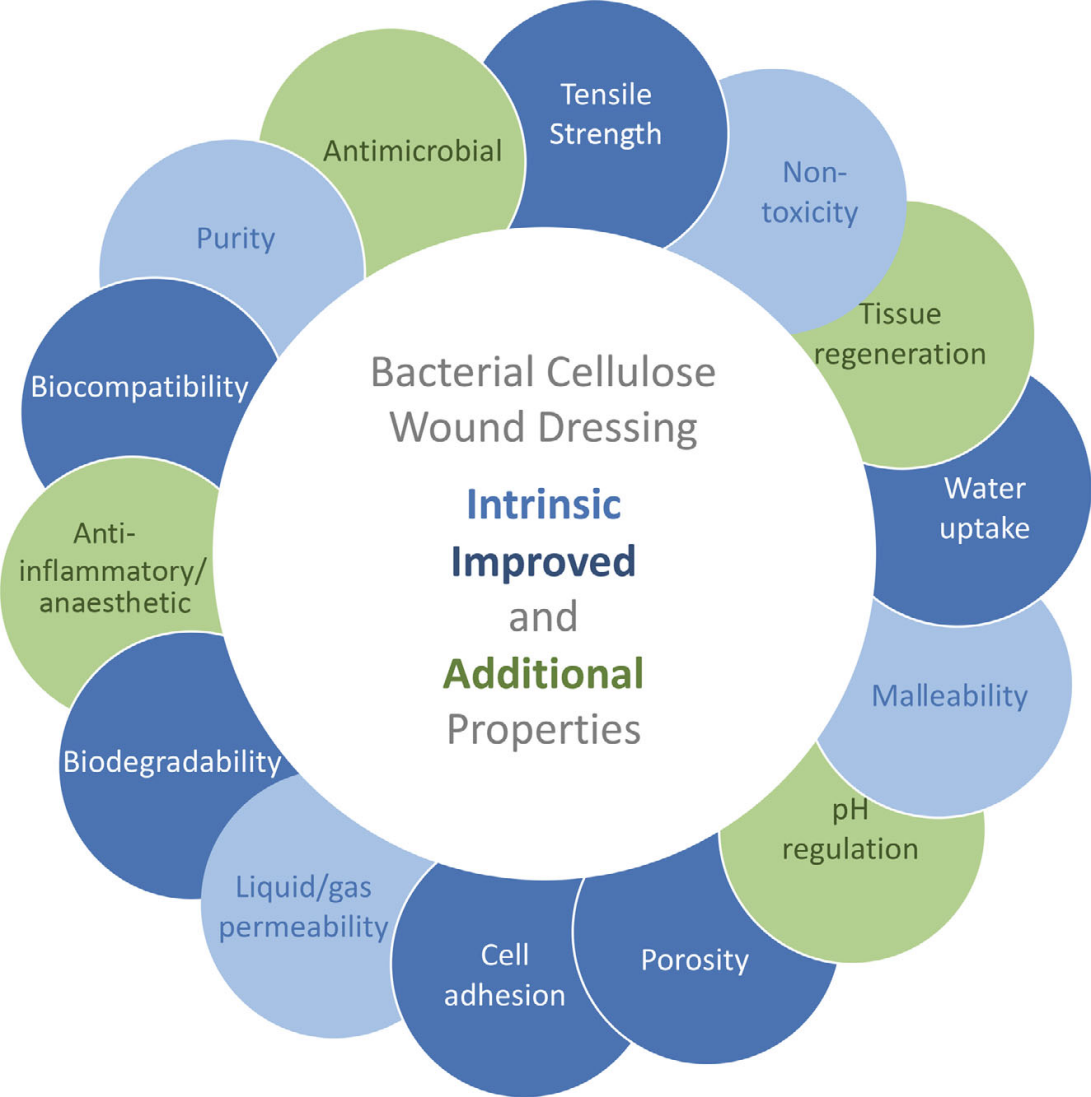
Main improvements of bacterial cellulose for wound dressing applications. Light blue – intrinsic properties of BC that present advantages for wound dressing applications. Dark blue – intrinsic properties of BC that suffered improvements. Green – additional properties that were introduced into BC for wound healing improvement.

BC has drawn attention in applications such as a wound dressing material, due to its intrinsic properties, namely its hydrophilicity, very high purity, porosity, biocompatibility and, since it also exhibits a network structure, controlled drug release capability that can be availed. In order to improve its efficiency as a wound dressing material or to provide it with tailored properties or functionalities, strategies have relied on the exploitation of its natural properties and improvement as in the case of tensile strength, biocompatibility and water uptake, among others. Nevertheless, BC alone does not present several desirable characteristics, such as antibacterial activity or anti‐inflammatory properties. In this way, in addition to the improvement of the natural features, new properties have been introduced into BC, mainly through the development of BC composites (Fig. 4). In Table 1, a summary of examples of BC composites is presented in which the reinforcement meets the wound dressing function that was intended to be enhanced.

**Table 1:** Examples of BC composites and the respective improved properties

BC reinforcement	Improved Function	References
Poly(vinyl alcohol) (PVA)	Enhancement of the mechanical performances	Castro *et al*. (2015) and Qiao *et al*. (2015)
Dehydrogenative polymer of coniferyl alcohol (DHP)	Improving the antibacterial activity	Zmejkoski *et al*. (2018)
Silver nanoparticles	Improving the antibacterial activity	Volova *et al*. (2018), Tabaii and Emtiazi (2018), Shao *et al*. (2016a,2016b), Wen *et al*. (2015), Wu *et al*. (2018) and Pal *et al*. (2017)
Chitosan and alginate	Higher elongation, rehydration, swelling ratios and water vapour transmission	Chang and Chen (2016)
Hyaluronan	Improving the thermal stability, lower total surface area and pore volume, weight loss and elongation at break	Li *et al*. (2014a,2014b, 2015)
Acrylic acid	Promoting faster wound healing, enhanced epithelialization and accelerated fibroblast proliferation	Mohamad *et al*. (2014a,b)
Zinc oxide (ZnO)	Improving the antibacterial activity	Janpetch *et al*. (2016)
Arginine	Promoting proliferation, migration and expression of collagen‐I of fibroblasts and endothelial cells	Qiao *et al*. (2018)
Antibiotics	Improving the antibacterial activity	Volova *et al*. (2018), Shao *et al*. (2016a,2016b), Lazarini *et al*. (2016)
Magnetic nanoparticles (magnetite)	Improving the efficiency of chronic wounds healing	Galateanu *et al*. (2015)
Agarose	Improving the mechanical properties and water uptake	Awadhiya *et al*. (2017)
Titanium dioxide (TiO_2_)	Promoting healing and tissue regeneration	Khalid *et al*. (2017) and Khan *et al*. (2015)
C_60_ nanoparticles	Improving skin cancer wound therapy	Chu *et al*. (2018)
Poly(lactic acid) (PLA)	Improving the mechanical properties	Foong *et al*. (2018)
BC bilayer with different fibre densities	Improving the controlled release of different antibiotics to treat skin infections.	Lazarini *et al*. (2016)
Gold nanoparticles	Improving the antibacterial efficiency	Li *et al*. (2017a,2017b)
Montmorillonites and silver nanoparticles	Improving the antibacterial efficiency	Li *et al*. (2017a,2017b)
Sodium alginate (SA)	Improving the mechanical properties	Lin *et al*. (2014)
Graphene oxide/silver nanohybrid	Improving the antibacterial efficiency	Mohammadnejad *et al*. (2018)
Plasticizers (PEG and Glycerol)	Improving the physicochemical properties	Sun *et al*. (2018)
Hydrolysed gelatin peptide	Improving the physicochemical properties	Lin *et al*. (2015a,2015b)
Chitosan	Improving the physicochemical and antibacterial properties	Savitskaya *et al*. (2017), Zhang *et al*. (2016)

### Physical properties’ optimization

In order to be used as a wound dressing material, the robustness of the BC films is a key issue that has driven efforts to improve their mechanical properties. Qiao *et al*. (2015) were able to produce a regular and uniformly distributed porous structure with enhanced mechanical properties, through the interaction of BC nanofibres with the PVA polymeric molecules, forming physical cross‐linked composite hydrogels. Chitosan (Ch), N‐deacetylated derivate of chitin, is a natural polysaccharide exhibiting exceptional physicochemical properties such as vapour permeability, antibacterial activity, biocompatibility and outstanding film‐forming capability. When chemically decomposed, chitosan releases N‐acetyl‐β‐d‐glucosamine instigating fibroblast proliferation and controlled collagen deposition, resulting in a quicker wound healing. Nevertheless, films produced from pure chitosan lack on mechanical robustness (brittle) and the cost of chitosan is relatively high, limiting its application.

Chang and Chen (2016) produced a chitosan and alginate BC composite, after treating the BC with hydrogen peroxide, that not only exhibited the desirable mechanical properties but also presented rehydration properties which enabled the usage of these composites as wound dressing materials for exudate absorption and the eventual controlled drug release. Another chitosan composite was proposed by Savitskaya *et al*. that they modify the BC by immobilization of chitosan, resulting in a composite material containing glucosamine and N‐acetylglucosamine units integrated into the cellulose chain. These composites presented challenging properties such as good mechanical properties, high moisture‐retaining properties and high antibacterial activity against gram‐negative and gram‐positive bacteria. These qualities make BC/Ch composite a candidate not only to be used as a wound dressing material but also for tissue engineering (Savitskaya *et al*., 2017).

Mohamad *et al*. developed and characterized BC/acrylic acid hydrogels with the purpose of improving the BC wound healing potential. These composites presented a macroporous network structure having high swelling ratio and high water vapour transmission rate which are important properties in terms of absorbing exudates and providing hydration for healing in particular burn wounds (Mohamad *et al*., 2014). By dissolving different amounts of magnetite nanoparticles throughout the biosynthesis process, BC composites were produced straight from the BC culture medium. It was proven that the existence of magnetite nanoparticles during the biosynthesis process does not disturbs it. Using this technique, and producing wound dressings from BC/magnetite composites, it was possible to improve the physical, chemical, morphological and biological properties of pure BC (Galateanu *et al*., 2015). Besides its antibacterial activity, silver nanoparticle composites proved to increase the physical properties of pure BC. The silver nanoparticles can be synthetized inside the porous three‐dimensional BC structure; this network is then irradiated with UV light so that the silver nanoparticles are photochemically deposited onto the BC hydrogel. The silver nanoparticles stay chemically bonded to the cellulose fibre surfaces, presenting a narrow size distribution along the BC. Since the composite pellicles are conserved in a moist environment, the wound healing is more efficient (Pal *et al*., 2017). Agarose is a biodegradable polymer with limited mechanical robustness and excessive water uptake. These properties limit its usage as wound dressing but when used in a composite with for instance BC, very challenging properties can be achieved. BC/agarose composites proved to present good mechanical and swelling properties, thermal stability and biodegradability, making them suitable as wound dressing materials (Awadhiya *et al*., 2017). Poly(lactic acid) (PLA) has been suggested as coating material (concentrations below 10%). This layer exhibits low moisture uptake, prolonged swelling simulated body fluid, high tear and burst indices. Foong *et al*. demonstrated that incorporating 8% of PLA on BC makes the composite more suitable to use as a wound dressing with antimicrobial properties. Using a BC/PLA composite, they were able to improve the mechanical properties, maintaining a reasonable wetting time. Also, they observe a preferable surface morphology on a microscopic level (the PLA coating changed into a more fibrous and porous morphology) with a low moisture uptake and prolonged swelling behaviour in simulated environment (Foong *et al*., 2018).

Alginate is a biomaterial that was already applied in several biomedical applications due to its profitable properties, such as biocompatibility and ease of gelation. Lin *et al*. presented a BC/sodium alginate (SA) composite having an interpenetrating polymer network structure. This composite presented outstanding swelling ratios, tensile modulus, tensile strength and elongation when compared with pure BC. This study confirmed that the interpenetrating structure radically changes the swelling and mechanical properties of the composite and enables it as a promising candidate for biomedical applications as wound dressings and skin tissue engineering (Lin *et al*., 2014). It is known that dried BC possesses poor gas permeability and water absorption. In order to improve dry BC physicochemical properties, Sun *et al*. performed comparative studies using two biocompatible plasticizers with different molecular weight and hydroxyl content, glycerol (G) and polyethylene glycol (PEG). They demonstrated that glycerol and PEG did not only cover the BC microfibres but also expanded the free space among the fibres, creating a highly porous structure. The toughness of the composites was efficiently increased when compared with pure BC, and the water absorption/retention capabilities of the BC composites were considerably higher than dry BC. Furthermore, the highly porous structure formed with the plasticized dry BC composites perfected its water vapour transmission. The plasticized dry BC composites also exhibited excellent resistance against bacteria (Sun *et al*., 2018).

### Biological functions’ optimization

Although having outstanding physical and chemical properties as scaffolds for wound dressing applications, BC native characteristics are not enough to meet the current needs in the dressing material market. Nowadays, it is expected from a dressing material that it has a functional contribution in the healing process. The major complications that frequently arise include the contamination with opportunistic pathogens and subsequent development of infection and inflammation and also the development of tumours that contribute to the development of chronic wounds (Fonder *et al*., 2008).

The rapid emergence of antibiotic resistance among a high number of bacterial pathogenic species (World Health Organization, 2015) poses an additional problem for patients with chronic or severe burn wounds that are frequently and recurrently hospitalized. The scenario of an open wound is beneficial for nosocomial agents, usually multi‐drug‐resistant strains, that rise due to selective pressure characteristic of healthcare facilities. Bacterial infections are in fact the most common clinical complication, usually associated with skin conditions and play a pivotal role in treatment failure or delay of the healing process, causing patient distress and financial burden. Colonization with bacteria is especially critical in burn wounds since these patients usually have compromised immune systems and a wide disruption of the skin barrier (Calum *et al*., 2009).

Though BC provides a physical barrier that reduces bacterial penetration into the tissues, in its native form it does not present antimicrobial properties *per se* (Czaja *et al*., 2006). To improve its efficiency as a therapeutic agent for treatment or for prophylactic purposes, modifications have been introduced to BC structure or specific compounds were added, to confer diverse biological activities, such as antimicrobial or anti‐inflammatory, to BC wound dressings.

Different approaches have recently been adopted to develop topical functionalized wound dressings with altered composition. Compounds that were described to have been incorporated into BC, at stage of development or preclinical tests, include not only small molecules but also macromolecules and complex polymers. Three main compound‐loading strategies have been used so far, post‐synthesis loading by saturation, by chemical modification of the purified BC structure or through genetic engineering approaches. The choice of the incorporation strategy depends on the physicochemical characteristics of the active compound, such as molecular size, solubility, stability and working concentration, on the type of BC network, like native wet, semidried or freeze‐dried, and also on the bacterial strain used as producer. Importantly, the functionalization method will influence the time‐release rate of the compound.

Lignin‐derived compounds can be used as antibacterial reinforcement in BC composites in order to improve the antibacterial action of the wound dressings. Zmejkoski *et al*. (2018) used a lignin model polymer (dehydrogenative polymer of coniferyl alcohol) to produce BC composite hydrogels presenting a decrease in the pore number and size and, due to its antimicrobial action, a faster skin repair and decrease of pain in patients. Also, in recent years, due to the emergent threat of bacterial resistance to antibiotics, alternatives such as silver nanoparticle composites have drawn scientific attention. Several types of silver nanoparticles can be employed such as silver sulfadiazine (Shao *et al*., 2016b) or for instance silver nitrate (Tabaii and Emtiazi, 2018; Wu *et al*., 2018). These composites displayed excellent antibacterial performances for the most common human pathogens maintaining a good biocompatibility (Wu *et al*., 2018). The BC/silver nanoparticle composites are proven to be non‐toxic and exhibited good biocompatibility on peripheral blood mononuclear cells due to the controlled silver ion release (Tabaii and Emtiazi, 2018). These composites, containing silver nanoparticles, are transparent, allowing uninterrupted visualization of the wound without having to remove the dressing (Tabaii and Emtiazi, 2018). A comparative study performed on rat models demonstrated that the wound treated with pure BC containing silver nanoparticles presented greater healing rate when compared with BC, proving that these composites are very promising as wound dressing for burns (Wen *et al*., 2015). Volova *et al*. (2018) tested for the same purpose the usage of silver nanoparticles and antibiotics (amikacin and ceftriaxone), achieving a strong inhibitory effect on pathogens, without hindering the growth of epidermal cells. Other composites have been tested incorporating antibiotics, such as tetracyclines for improvement of the antibacterial activity (Shao *et al*., 2016a). It was stated by Lazarini *et al*. that BC produced in all culture media displays an intrinsic composite formed by a double layer (with different fibre densities) and three‐dimensional fibre network achieved in only one step. This 3D network structure of the bilayer with high‐density fibre entangling, produced in sugarcane molasses medium, is responsible for the greatest holding capacity and sustained release of the antibiotics such as ceftriaxone, used in the case of *Staphylococcus aureus* bacterial strains (Lazarini *et al*., 2016). Gold nanoparticle BC composites were said to present better efficacy than most of the antibiotics against gram‐negative bacteria, while preserving outstanding physicochemical properties such as water uptake capacity, high mechanical strain and biocompatibility. The broad antibacterial spectrum of these composites along with the desirable moisture retention and the good mechanical properties enables them as an excellent material for wound dressing (Li *et al*., 2017b). TiO_2_ nanoparticles are known for their super‐hydrophilic, chemical stability and biocompatibility (Fujishima *et al*., 2000). *In vivo* wound healing efficacy of BC/TiO_2_ composites was assessed in a burn wound model by measurements of wound area, per cent contraction and histopathology. The results showed that BC/TiO_2_ composites acquired an outstanding healing potential presenting faster re‐epithelialization degree as well as enhanced wound contraction capability (Khalid *et al*., 2017). In addition to antibacterial properties, Khan *et al*. (2015) demonstrated that the BC/TiO_2_ composites exhibit remarkable cell adhesion and proliferation capabilities with animal fibroblast cells without displaying any toxic effects. Janpetch *et al*. studied BC/zinc oxide composites for antimicrobial activity enhancement. Zinc oxide (ZnO) is known as an inorganic antibacterial agent and BC proven to be an excellent upholding template for the coordination of ZnO. They demonstrated that the ZnO content in these composites is determinant to improve the disinfection capabilities of BC (Janpetch *et al*., 2016). Qiao *et al*. studied BC/arginine composites after oxidizing the BC with a novel technique. High oxidation degree of BC increases the amount of aldehyde, which reduces the cell biocompatibility of BC. Using this new method to oxidize BC and producing arginine composites, they increased the roughness and surface energy of BC and were able to stimulate the propagation, migration and expression of collagen‐I of fibroblasts and endothelial cells (Qiao *et al*., 2018). Using photodynamic therapy, BC/C_60_ can be used as wound dressings for skin cancer treatment. The C_60_ particles, homogeneously distributed in the 3D BC network, proved to possess a high capability to generate reactive oxygen species under light exposure and so inhibit the growth of several bacteria. BC/C_60_ composites presented low cytotoxicity in the dark; however, they demonstrated substantial cancer cell destruction when exposed to visible light (Chu *et al*., 2018). Li *et al*. presented smaller‐sized silver nanoparticles evenly immobilized in montmorillonites, which gave rise to BC composites demonstrating high antimicrobial activity. Besides owing the desirable mechanical and hydrophilic properties, these composites revealed low silver release. Even though the silver release ratio was short, the small particle size of the silver nanoparticles allowed them to more effectively penetrate the bacterial cells and possess high electrostatic affinity to interrelate with the cell membrane to obstruct bacterial growing (Li *et al*., 2017a). Graphene oxide–silver nanohybrid was used to confer BC antibacterial activity by producing a BC/graphene oxide–silver nanohybrid composite. Mohammadnejad *et al*. (2018) demonstrated that the presence of graphene oxide–silver nanohybrid increased the mechanical strength and antibacterial activity of BC.

### Compound loading by immersion

Directly related to its structural properties, as its microporous structure, large surface area and moisture retention capacity, BC is able to absorb and retain large amounts of active compounds. In the same way, these features of BC allow for the slow release of the compounds into the affected tissue and thus a more prolonged effect.

The loading of purified BC by submersion and saturation is the most frequent choice of compound incorporation, since the procedure is of simple implementation, although time‐consuming. The most reported strategy consists of soaking dried or semidried BC in solutions of the active compound. Through this method has been described the functionalization of BC with antiseptic compounds, such as octenidine, povidone‐iodine (PI) and polyhexanide (PHMB) (Table 2) (Moritz *et al*., 2014; Wiegand *et al*., 2015; Alkhatib *et al*., 2017), and the release of all these compounds relied on diffusion and swelling. Octenidine loading did not affect the tensile strength of the BC matrix that presented a biphasic release profile, as the release rate was faster during the first 8 h and subsequently decreased up to 96 h. Due to its high molar mass, functionalization with PI introduced structural changes in the BC matrix that increased its compressive strength while incorporation of PHMB did not alter the tensile properties of BC. In accordance, PI showed a slower release process in comparison with PHMB. All antiseptic composites showed high biocompatibility in human keratinocytes but different antimicrobial activity against *S. aureus*, being PI the less active compound.

**Table 2:** Examples of incorporated biologically active agents in BC for wound dressings

Incorporated agent		Incorporation strategy	Therapeutic purpose	References
*Antibiotics*				
Fusidic acid	Small molecule	Adsorption by immersion	Antimicrobial activity	Liyaskina *et al*. (2017)
Tetracycline	Small molecule	Adsorption by immersion	Antimicrobial activity	Shao *et al*. (2016a,2016b)
Amoxicillin	Small molecule	Chemical cross‐linking	Antimicrobial activity	Ye *et al*. (2018)
Erythromycin	Small molecule	Adsorption by immersion	Antimicrobial activity	Zywicka *et al*. (2018)
*Non‐steroidal anti‐inflammatory drug*				
Diclofenac	Small molecule	Adsorption by immersion	Pain and inflammation relief	Silva *et al*. (2014)
Ibuprofen			Pain and inflammation relief	Trovatti *et al*. (2012)
*Local anaesthetic*				
Lidocaine	Small molecule	Adsorption by immersion	Pain relief	Trovatti *et al*. (2012)
*Cationic antimicrobial agents*				
Octenidine dihydrochloride	Small molecule	Adsorption by immersion	Antimicrobial activity	Moritz *et al*. (2014)
		Incorporation through poloxamers micelles		Alkhatib *et al*. (2017)
Povidone‐iodine	Small molecule	Adsorption by immersion	Antimicrobial activity	Wiegand *et al*. (2015)
Polyhexanide (PHMB)	Macromolecule	Adsorption by immersion	Antimicrobial activity	Wiegand *et al*. (2015)
Benzalkonium chloride	Small molecule	Adsorption by immersion	Antimicrobial activity	Mohite *et al*. (2016)
*Peptides or proteins*				
Laccase		Adsorption by immersion (two‐step method)	Antimicrobial activity	Sampaio *et al*. (2016)
Silk sericin		Adsorption by immersion	Re‐epithelialization (increases collagen production)	Napavichayanun *et al*. (2015)
Lysozyme	Macromolecules	Adsorption to phosphorylated BC	Antimicrobial activity	Oshima *et al*. (2011)
ε‐poly‐l‐Lysine (antimicrobial peptide)		Covalent conjugation by carbodiimide chemistry	Antimicrobial activity	Fursatz *et al*. (2018)
*Cells*				
Mesenchymal stem cells				Bobis *et al*., 2006)
Adipose mesenchymal stem cells				Souza *et al*. (2014)
Rabbit bone marrow mesenchymal stem cells		Cell seeding	Promote tissue regeneration	Silva *et al*. (2018)
Human epidermal keratinocytes				Loh *et al*. (2018)
Dermal fibroblasts				Loh *et al*. (2018)
*Other*				
Berberine Isoquinoline alkaloid	Small molecule	Adsorption by immersion under boiling	Antibacterial, anti‐inflammatory, antitumour	Huang *et al*. (2013)
Quaternary ammonium compounds	Small molecule	Adsorption by immersion	Antimicrobial activity	Zywicka *et al*. (2018)
Arginine	Small molecule	Grafting to oxidized BC	Re‐epithelialization (increases collagen production)	Qiao *et al*. (2018)

Antibiotics have also been loaded into BC by immersion as the case of tetracycline, a short‐acting broad‐spectrum drug that inhibits bacterial growth by inhibiting translation. Loading was performed during 24 h with gentle stirring which resulted in a denser BC network structure and, after an initial burst release, the BC composite displayed a steady release of tetracycline (Shao *et al*., 2016a). Recently, saturation of BC with fusidic acid, a steroid antibiotic usually used for topic applications through a cream or eyedrops, was performed by simple immersion of the BC films for 1–24 h, but the release rate was not monitored. The resulting membrane was shown to be active against *S. aureus* (Liyaskina *et al*., 2017). The BC/antibiotic composite membranes presented excellent biocompatibility and effective antibacterial activity against gram‐negative and gram‐positive species.

Benzalkonium chloride, an antimicrobial cationic surfactant effective against gram‐positive bacteria, widely used in commercial wound dressings, was tested for BC loading by overnight soaking. The drug‐loading capacity increased with the drug concentration, and the release rate was described to be of 90% of the drug within the first 24 h. The cytotoxicity was tested on human peripheral blood mononuclear cells with 90% cell viability, which allows its application as a regenerative biomaterial (Mohite *et al*., 2016).

Some variations of the loading process have been reported, such as vortexing of wet BC for protein loading. In comparison with conventional methods, vortexing for a 10‐min period resulted in the same protein loading level than submersion for 24 h. While protein distribution and stability were unaltered, vortex loading resulted in a much slower protein release, directly related to a denser BC matrix and reduced capacity of water holding (Muller *et al*., 2014). Another variation was performed by boiling the purified BC membrane in a solution of berberine, an isoquinoline alkaloid extracted from Chinese medicinal herbs with several therapeutic activities such as antimicrobial, anti‐inflammatory and antitumour, among others. Although the authors pretended to use BC membranes as controlled release systems for ingestion and survival to gastric fluids, release studies and transdermal assays showed that BC significantly extends berberine release time and the results were transposed to skin delivery applications. The lowest release rate observed for BC/berberine composite was for acidic conditions, such as the skin, in simulated gastric fluid or in H_2_SO_4_ solution, the highest rate was in simulated intestinal fluid, and an intermediate rate was found in alkaline conditions. This behaviour was found to be directly related to the pore size of the BC matrix, as the pore size decreased after treatment with NaOH due to swelling of the BC fibres, hindering the diffusion of the drug from the pores of BC. This study showed that besides the factors already described in the literature to influence drug release from BC, the external environment also plays a major role and must be considered. Interestingly, solid‐state NMR assays revealed an interaction between berberine and the structure of BC (Huang *et al*., 2013).

Hyaluronan, a glycosaminoglycan present in the synovial fluid that enwraps joints, cartilage and tissues, allows the binding of a large number of water molecules, improving tissue hydration. Also, its rheological properties increase fluid viscosity, providing tissue resistance to mechanical damage. Hyaluronan is known for its curative characteristics, associated with pro‐angiogenic and anti‐apoptotic properties, endorsing the recovery of wound skin tissue and decreasing scar formation (Li *et al*., 2015). Depending on its molecular size, it can have anti‐inflammatory and immunosuppressive effects – high molecular weight hyaluronan – or have pro‐inflammatory action – low molecular weight hyaluronan (Litwiniuk *et al*., 2016). In fact, during the skin repair process, a rapid increase in hyaluronan is associated with tissue swelling, epithelial and mesenchymal cell migration and proliferation, and induction of cytokine signalling. Hyaluronan extending from cell surface into structures called cables can trap leucocytes and platelets and change their functions, modulating inflammation. Li *et al*. studied the physical properties of BC composites containing high molecular weight hyaluronan as stimulus on the healing process. These BC composites, obtained through a solution impregnation method, presented enhanced properties in terms of thermal stability, lower total surface area and pore volume, weight loss and elongation at break (Li *et al*., 2014a).

Lidocaine, used as local anaesthetic, and ibuprofen, a common use non‐steroidal anti‐inflammatory drug, were chosen as model hydrophilic and hydrophobic compounds, respectively, for the development of topical BC drug delivery systems. While a lidocaine aqueous solution was used to soak previously drained BC membranes, in the case of ibuprofen, the water of the BC membrane was previously replaced by ethanol. Subsequently, these pre‐treated membranes were immersed in an alcoholic ibuprofen solution. Diffusion assays showed a lower release rate for lidocaine but a 3 times higher release rate for ibuprofen, than in commercial dressings (Trovatti *et al*., 2012). More recently, the loading of diclofenac, a non‐steroidal anti‐inflammatory compound, frequently used to relieve pain and inflammation in short‐term clinical situations, was performed by immersion of drained BC membranes in a diclofenac solution with 5% glycerol as plasticizer. The BC‐diclofenac membranes presented a 6 times higher swelling behaviour and a release rate much slower than commercial gels, suggesting BC membranes as an advantageous transdermal delivery system for diclofenac (Silva *et al*., 2014).

Quaternary ammonium compounds (QACs) are low molecular weight biocides that include a positive charge and a hydrophobic segment. They present high cell membrane penetration capacity, low toxicity and antibacterial activity, dependent on the chain length of the alkyl chain. Recently, a QAC was synthesized by coupling reaction between the C18 long‐chain unsaturated fatty acid, the dimer dilinoleic acid (DLA), tyrosine and positively charged ethylenediamine (Umeda *et al*., 1999). The resulting compound [EDA]‐[DLA‐Tyr] was loaded by immersion for 24 h on BC membranes and showed antimicrobial activity against *S. aureus* and *S. epidermidis*, both opportunistic pathogens highly associated with skin and wound infections, particularly in hospital settings such as surgical or indwelling device‐associated wounds.

Regarding enzyme immobilization, the choice of a suitable carrier is mandatory, since the procedure cannot impair the activity of the enzyme. Although most carriers need to be activated before immobilization, resulting in low efficiencies, BC membranes can be loaded by simple soaking, not affecting the enzyme correct folding. The incorporation, by immersion, of proteins into BC membranes for the development of delivery systems has been analysed using model proteins, such as serum albumin. The loading was optimized by using never‐dried, pre‐swelled BC membranes, which was due to alterations of the fibre network during the freeze‐drying process, and the biological activity of the proteins was maintained during the loading and release steps (Muller *et al*., 2013). Another variation strategy to the method of protein loading was assayed for lipase model protein, a strategy designated as repeated absorption, a two‐step method involving repeated drying and absorption and activation with glutaraldehyde‐reticulating agent. The limit solution was forced into dried BC during absorption and the enzyme immobilization efficiency was higher than 90%, for the different two‐step absorption methods tested. The immobilized lipase retained 60% of its native activity after 15 repeated usages, suggesting that the two‐step immobilization method of enzymes is suitable for industrial applications (Wu *et al*., 2016). The immobilization of an enzyme with a therapeutic application was performed for laccase, due to its antibacterial activity. The loading of the enzyme was done by immersion of BC membranes, and the specific activity of the immobilized enzyme did not differ much from that of the free enzyme. The entrapment process maintained some flexibility degree and even improved access to the substrate, resulting in high antimicrobial activity for gram‐positive bacteria and a cytotoxicity level acceptable for wound dressing applications (Sampaio *et al*., 2016).

Also, conjugated strategies have added more than one different activity to the BC matrix, through adsorption loading approaches. One recent example involved the incorporation by immersion of two components into BC, silk sericin to enhance collagen type I production, which is critical for re‐epithelialization and the antiseptic PHMB. The interactions between these two components were analysed, and it was observed that silk sericin needed to be loaded before PHMB to maintain PHMB antimicrobial activity against all tested bacteria (*Bacillus subtilis, S. aureus,* methicillin‐resistant *S. aureus, Escherichia coli, Acinetobacter baumannii* and *Pseudomonas aeruginosa*) (Napavichayanun *et al*., 2015, 2016). These type of approaches open new paths for the future incorporation of more than one valence into the same wound dressing.

### Compound loading by modification

Although more rare than the immersion techniques, other strategies have been adopted to load active compounds into BC. Despite being more complex, expensive and time‐consuming, they can present advantages such as controlled release and increased activity. Chemical modifications of the BC composition, allowing for the immobilization of compounds or proteins, have enhanced the interaction between the two components. BC presents a large amount of exposed hydroxyl groups that can be functionalized through different approaches.

Different adsorption strategies have been tested for proteins, namely lysozyme and as model proteins, haemoglobin, myoglobin and albumin. The BC modifications that resulted in promising adsorption results open new routes for BC functionalization with a wide range of enzymatic activities of therapeutic interest in wound healing. In a phosphorylation approach, BC was phosphorylated with phosphoric acid in the presence of *N*,*N*‐dimethylformamide (DMF) and urea at various degrees. The adsorption capacity for lysozyme increased with the percentage of BC phosphorylation and was much higher than that of plant cellulose (PC), since the specific surface area of phosphorylated BC is much higher than that of phosphorylated PC. In fact, the adsorption capacity of small molecules was similar for both types of phosphorylated cellulose, since these can more easily access internal adsorption sites (Oshima *et al*., 2008, 2011). In the surface carboxymethylation approach, the hydroxyl groups of BC suffered chemical substitution by treatment with NaOH followed by addition of ethanol and chloroacetic acid. The adsorption of albumin to carboxymethylated BC occurred at pH values below its isoelectric point by electrostatic interaction and increased with the degree of substitution (Lin *et al*., 2015a).

In another modification strategy, quaternary ammonium groups were introduced into BC as an adsorption approach for proteins. This strategy did not produce alterations in the microfibrous structure, and the modified BC showed selectivity for proteins over other compounds and higher adsorption capacity than PC with the same modification. The model protein haemoglobin was adsorbed on the quaternary ammonium BC under pH conditions lower than its isoelectric point, via electrostatic interactions (Niidei *et al*., 2010).

Recently, controlled release studies were performed to develop a long‐term dermal wound dressing. While octenidine had previously been shown to be stable, releasable and biologically active for over 6 months storage, the drug release time window was approximately 96 h. The new approach involved the modification of BC by incorporation of poloxamers as micelles and gels and resulted in prolonged retention time of octenidine up to 1 week together with upgraded mechanical and antimicrobial properties (Alkhatib *et al*., 2017).

The association of amoxicillin, a β‐lactam antibiotic, with BC sponges was performed by a cross‐linking coupling strategy. The BC was pre‐treated with 3‐aminopropyltriethoxysilane (APTES) in order to graft aminoalkylsilane groups through Si‐O‐C bonds. The amoxicillin was also modified at the carboxylic reactive group by treatment with the carbodiimide cross‐linker EDC/NHS (1‐ethyl‐3‐(3‐dimethylaminopropyl)carbodiimide hydrochloride). The NHS‐activated ester groups of amoxicillin are then able to react with the terminal NH_2_ groups of BC, resulting in covalent links between amoxicillin and BC. The BC/amoxicillin graft increased the antimicrobial activity against *S. aureus*,* E. coli* and *Candida albicans* and showed good cytocompatibility (Ye *et al*., 2018).

Functionalization of BC was performed for ε‐poly‐l‐Lysine (ε‐PLL), an antimicrobial peptide with broad‐spectrum antimicrobial activity that belongs to the first line of the innate immune system of many organisms. Besides being non‐toxic, water‐soluble and biodegradable, its mechanism of action, disruption of the bacterial cell membrane, diminishes the hypotheses of resistance emergence. Low molecular weight ε‐PLL (~4–5 kDa) was functionalized into BC following two different strategies. In the first, ε‐PLL was covalently conjugated through carbodiimide chemistry to previously carboxymethyl‐functionalized BC membranes. In the second, ε‐PLL was directly cross‐linked with the BC structure using carbodiimide chemistry. Both strategies resulted in membranes with unaltered cytocompatibility to human fibroblasts and with capacity to inhibit growth of *S. epidermidis* on contact. The functionalization with ε‐PLL had no significant effects on the nanofibrous structure and mechanical properties of BC (Fursatz *et al*., 2018).

Charged BC derivatives, carboxylated and aminated forms, were obtained by 2,2,6,6‐tetramethylpiperidine‐1‐oxyl radical (TEMPO)‐catalysed oxidation reaction and by the epichlorohydrin‐mediated amination reaction. These BC derivatives showed interesting properties for drug delivery via ionic conjugation, since both the cationic and anionic forms showed increased water retention capacity in a pH‐responsive way (Spaic *et al*., 2014). Similarly, a recent BC composite was developed using oxidized BC of microporous structure that provides a higher contact area. Arginine was grafted into the oxidized BC and, besides showing enhanced biocompatibility, also promoted collagen synthesis (Qiao *et al*., 2018).

Several types of BC/metal nanocomposites were successfully developed and showed high levels of antibacterial activity. Berndt *et al*. used an incorporation approach of silver nanoparticles in BC by a stepwise modification of a method previously used for two‐dimensional cellulose films and now applied to a 3D structure. Usual methods use AgNO_3_ in combination with strong reducing agents, and the relatively large particle agglomerates formed are immobilized by physical interactions and not by chemical bonds. In this work, a mild chemical, dimethyl sulfoxide (DMSO) was used as a reducing agent to activate the BC membranes that were subsequently immersed in a solution of 1,4‐diaminobutane and finally in a solution of DMSO, sodium acetate and AgNO_3_. Appended amine groups operated as anchoring centres for the chemical immobilization of the AgNPs. The BC/AgNP chemical linkage showed increased retention time maintaining strong antimicrobial activity against *E. coli*, even for low amounts of AgNPs (Berndt *et al*., 2013).

More recently, modification of BC by TEMPO‐mediated oxidation was performed with TEMPO/NaClO/NaBr system to obtain anionic C6 carboxylate groups. The modified BC was then incorporated with silver nanoparticles (AgNP) by ion exchange in AgNO_3_ solution. The BC/AgNP membranes showed low cytotoxicity for a NIH3T3 fibroblast cell line (cell viability of 95.2 ± 3.0% after 48 h) and antibacterial activities of 100% and 99.2% against *E. coli* and *S. aureus* respectively (Wu *et al*., 2018).

## Incorporation of cells into bacterial cellulose

One of the most recent strategies to improve a BC wound dressing for the effective treatment of skin injuries is the incorporation of mesenchymal stem cells (MSC) in the matrix. MSC are adult pluripotent cells that can differentiate into a minimum of two cellular types (Bobis *et al*., 2006). These cells are expected to integrate into the host tissue and promote the regeneration of the damaged tissue.

**Figure 5:**
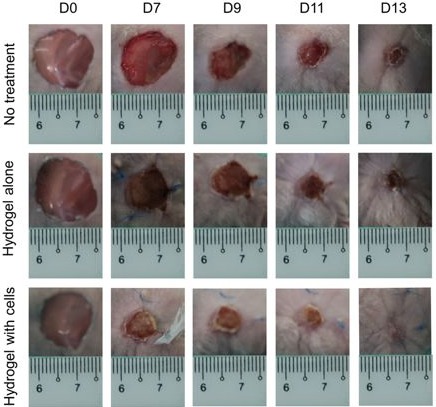
Healing process of wound excised (15 mm diameter) on a rat. Index *D*_0_ stands for the initial state, *D*_7_ for the 7th day, *D*_9_ for the 9th day, *D*_11_ for the 11th day and *D*_13_ for the 13th day. On the first row (No treatment), control wounds of the untreated group. On the second row (hydrogel alone) test, wounds of the group treated with a BC wound dressing. On the third row (hydrogel with cells) test, wounds of the group treated with a BC/cells wound dressing. Reprinted from Scientific Reports, Vol 8, 2875, E.Y.X. Loh, N. Mohamad, M.B. Fauzi, M.H. Ng, S.F. Ng, M.C.I.M. Amin, Development of a bacterial cellulose‐based hydrogel cell carrier containing keratinocytes and fibroblasts for full‐thickness wound healing, Copyright (2018), with permission under a Creative Commons Attribution 4.0 International License.

Several studies were performed in which MSC, multipotent cells that can differentiate into numerous cell types, including bone, cartilage, muscle and fat cells, were added to BC membranes. MSC have a great capacity to self‐renew, while maintaining its multipotency, an essential feature to improve the process of wound healing and inducing re‐epithelialization of the wound. In one example, adipose MSC (AMSC) were obtained from human adipose tissue liposuction and incorporated into BC membranes. The BC/AMSC membranes were then tested in rats with induced burns. The results showed that the AMSC differentiated into adipocytes and osteocytes with a high regenerative potential (Souza *et al*., 2014). Another example used rabbit bone marrow MSC (BM‐MSC) associated with BC (BC/BM‐MSC). The BM‐MSC were observed, by scanning electron microscopy (SEM), to have fully integrated within the BC matrix and to have the ability to differentiate into more than one mesenchymal lineage (chondrogenic, osteogenic or adipogenic) once integrated into the matrix, yielding membranes with good biocompatibility results (Silva *et al*., 2018). Besides stem cells, other types of cells were added to BC membranes; human epidermal keratinocytes and dermal fibroblasts (DF) were incorporated in a BC/acrylic acid (AA) hydrogel exhibiting wound healing ability *in vitro* and in *in vivo* models (Fig. 5). The results showed that the EK and DF cells can be transferred to the wound and accelerated the wound healing process (Loh *et al*., 2018).

The incorporation of AMSC and BM‐MSC cells in the BC membranes was performed by simple seeding. The ED and DF were also seeded on the sterilized BC/AA hydrogel pre‐soaked in the culture medium overnight.

## Genetic manipulation of bacterial cellulose

Genetic engineering of the BC producing bacteria has been explored with the aim to optimize the intrinsic properties of BC and the cost‐effectiveness of the production process. Strain improvement has been performed through the transfer of BC‐related genetic determinants to a previously prepared ‘cell factory’ organism, resulting in the heterologous expression of genes, or through the genetic reprogramming of the natural BC producers.

Examples of genetic improvement of BC include the transfer of genes *cmc, ccp, cesAB, cesC, cesD* and *bgl* from *K. xylinus* to *Synechococcus* sp., resulting in an increase in BC production, especially upon low salinity conditions (Zhao *et al*., 2015). Also, the simultaneous expression of the *bcs*ABCD operon and its upstream genes, *cmc*ax and *ccpAx*, was performed in *E. coli* (Buldum *et al*., 2018). The *cmc*ax genes encode for a BC‐hydrolysing enzyme, and *ccpAx* encodes for a protein related to the extrusion of the cellulose fibres (Wong *et al*., 1990) (Saxena *et al*., 1994) (Sunagawa *et al*., 2013). BC biosynthesis was detected earlier in the fermentation process and presented denser fibres than with *K. hansenii*. The heterologous expression of the *bcsD* gene from *K. xylinus* in a BC producer *E. coli* strain was shown to improve the crystallinity of the BC without altering the yield (Sajadi *et al*., 2017).

Regarding heterologous expression in the natural producer, the *Vitreoscilla* haemoglobin‐encoding gene *vgb* was expressed in *K. xylinus* improving BC production yield (Liu *et al*., 2018a). A chemical and physical random mutagenesis strategy was applied to *K. hansenii* and the mutants selected for presented low accumulation of organic acids, which is directly related to a higher BC production (Shigematsu *et al*., 2005). The accumulation of organic acids, by‐products of fermentation, competes with BC for carbon source utilization reducing its synthesis (Li *et al*., 2016).

To overcome the poor degradability of BC *in vivo*,* K. xylinus* was engineered to incorporate genes from *Candida albicans* to synthesize N‐acetyl‐glucosamine (GlcNAc) during BC synthesis, generating a modified cellulose that contains both glucose and GlcNAc. This altered BC structure showed susceptibility to lysozyme, a peptidoglycan hydrolytic enzyme that is abundantly produced in human secretions and by macrophages and polymorphonuclear neutrophils. This BC modification allows the development of wound dressings that can be degraded by the patient system, an advantage especially for burn wounds (Yadav *et al*., 2010).

The BC producer *K. xylinus* was also transformed with the curdlan synthase gene to produce biocomposites of cellulose and curdlan, an extracellular polysaccharide widely used in biomedical applications due to its low toxicity and non‐ionic gelation properties. This allowed the production of a pellicle of BC/curdlan, altering the pellicle's morphology and eliminating its pores without modifying the crystalline structure of BC (Fang *et al*., 2015).

A recent study used a sRNA interference system to control the native cellulose production path of the natural BC producer *K. rhaeticus*. The major achievements were the shut‐off of the constitutive BC production in order to prevent defective mutants to arise, a common phenomenon in well‐aerated conditions. Additionally, expression vectors were constructed to functionalize BC with specific proteins, by fusing the genes encoding the proteins of interest to short cellulose binding domains (Florea *et al*., 2016).

## Biocompatibility of bacterial cellulose

All the accepted definitions of biocompatibility rely on the capacity of a given material to meet its therapeutic functions once implanted in an animal host without triggering a local or systemic adverse reaction. A biocompatible material must meet several requirements that include tests related to cytotoxicity, sensitization, genotoxicity and carcinogenicity, among others. Usually, the first stage to pass is the low induction of an inflammatory response. BC has grown as a promising biomaterial for wound dressings, a role that requires filling the criteria of biocompatibility. BC holds relatively high scores of biocompatibility, a characteristic attributed to its nanofibrillar structure and to its purity, that allow the host cells to adhere and to proliferate.

Several studies, already reviewed, were conducted *in vitro* and *in vivo*, addressing the biocompatibility of BC in different forms (pellicles, membranes, and discs) and subjected to different treatments (NaOH and radiation), using cell lines or animal models within a time lapse that ranged from 1 week to 1 year (Sulaeva *et al*., 2015). Regarding the recent literature, the studies addressing BC biocompatibility increased in numbers and included a wide range of techniques. However, studies conducted in humans remain rare. One example was the study of Almeida and co‐workers that showed that BC used in the form of patches for 24 h did not promote skin irritation (Almeida *et al*., 2014). Also, BC tested for the treatment of chronic varicose ulcers of lower limbs for 120 days showed a decrease in the depth of the ulcer suggesting that BC induced tissue remodelling without associated toxicity (Cavalcanti *et al*., 2017).

Despite the low number of human trials, biocompatibility has been assayed using animal models and cell lines. A common technique to determine cytotoxicity is the MTT assay for the assessment of the cell metabolic activity, regarded as a measurement of cell viability and consecutively biocompatibility. The NADH‐dependent oxidoreductases that reduce the tetrazolium dye MTT 3‐(4,5‐dimethylthiazol‐2‐yl)‐2,5‐diphenyltetrazolium bromide to formazan are in a direct proportion to the number of viable cells (Berridge *et al*., 2005). This was used to assess the cytotoxicity of rabbit bone marrow mesenchymal stem cells (BM‐MSC) associated with BC in macrophages and showed a non‐toxic effect with 94% of cellular viability. In the same study, cytotoxicity was also assessed through the measurement of nitric oxide, produced by macrophages to eliminate pathogens, as an inflammatory response mediator, inhibiting or inducing inflammation. The colorimetric read of the NO released in the presence of the BC showed a non‐cytotoxic concentration (Silva *et al*., 2018).

Although toxicity determination can be reliably provided by indirect colorimetric methods, the effects of BC and of BC composites must be confirmed using cell lines and living animals. Chitosan is a promising agent for incorporation into BC because, once degraded by lysozyme, it releases mono‐ and oligosaccharides that stimulate angiogenesis (formation of new blood vessels) and tissue regeneration (Ishihara *et al*., 2006). In the context of regenerative medicine, a BC/chitosan composite, developed to treat hernias, was screened for biocompatibility using rats that were implanted with the BC/chitosan mesh. Histopathological examination of the organs and examination of the surrounding tissues searched for changes in the tissues and for the number and positioning of inflammatory cells. The same study also used rabbits to determine acute dermal irritation upon multiple dressing exchanges per day and to determine the intradermal reactivity through intracutaneous injections. No inflammation at the implant site was observed through histopathological analysis neither acute irritation nor allergic reactions. On the contrary, a higher degree of fibroplasia (the growth of fibrous tissue) was observed (Piasecka‐Zelga *et al*., 2018).

Although BC is recognized to have good biocompatibility with the different cells of the skin tissue, the adhesion of cells to BC in its native structure is not optimal. One factor that influences the response of the cells is the porosity of the mesh that can prevent the cell migration into the biomaterial. Usage of porogens, such as paraffin wax, to control the size of the pores was shown to lead muscle cells to be able to attach themselves inside the pores (Backdahl *et al*., 2008). In a recent study, the authors synthesized BC with the nanoporous structure altered to a microporous one. Gelatin microspheres were used both as porogens and as surface modification agents, eliminating the cytotoxic effects of other porogens and increasing its biocompatibility. Gelatin, a product of collagen hydrolysis, has been used for surface modification of polymeric scaffolds to mimic the composition of collagen in order to increase cell adhesion. The biocompatibility of the BC/gelatin with a microporous structure was determined in vitro using the HaCaT cell line as a keratinocyte model. Cells were able to proliferate and differentiate in the matrix, a required behaviour for tissue regeneration applications. Similar results were obtained in vivo through histological analysis of C57BL/6 (H‐2Kb) mice that suffered removal of a section of the dorsal flank skin. The process of re‐epithelialization was thus shown to occur in the BC/gelatin scaffold‐treated group with an increased healing effect and fewer inflammatory cells, compared with the control group (Khan *et al*., 2018).

Another study evaluated the biocompatibility of a hydrogel containing a mixture of carboxylated cellulose nanofibres (CNF) with aminated silver nanoparticles (Ag‐NH_2_ NPs) and gelatin (G). In vitro biocompatibility tests were performed with neonatal human dermal fibroblasts (NHDF) as a model of infected wounds. The viability of NHDF even increased due to the acceleration of the proliferation of the cells, induced by the matrix. In vivo wound healing testing was done on Kunming mice, and CNF/G/Ag showed a survival rate of 83%. However, the blood clotting tests showed good haemostatic properties that slightly decreased with the increase in Ag nanoparticles (Liu *et al*., 2018b).

Similarly, a recent biocomposite based on oxidized BC with microporous structure and in situ grafted with arginine that promotes collagen synthesis also showed enhanced biocompatibility (Qiao *et al*. 2018). The cytotoxicity of a BC wound dressing of TOBCP/AgNP, a BC pellicle that was modified by 2,2,6,6‐tetramethylpiperidine‐1‐oxyl radical (TEMPO)‐mediated oxidation and to which silver nanoparticles were incorporated for the antimicrobial activity of silver, was determined on a NIH3T3 fibroblast cell line, and the pellicle extracts presented a cell viability of 95.2 ± 3.0% after 48 h of incubation (Wu *et al*., 2018).

Mice models were used for the assessment of biocompatibility of BC with different pH values. An acidic pH at the wound site is described to promote faster wound healing, helping the proliferation of fibroblasts (Schreml *et al*., 2010). In this work, full‐thickness wounds were inflicted in the dorsal body surface of rats that subsequently received treatment with BC of different pH values (acidic, neutral and alkaline) and the evolution of the wound was monitored. The BC with acidic pH showed the best wound healing efficiency (Pourali *et al*., 2018).

**Figure 6:**
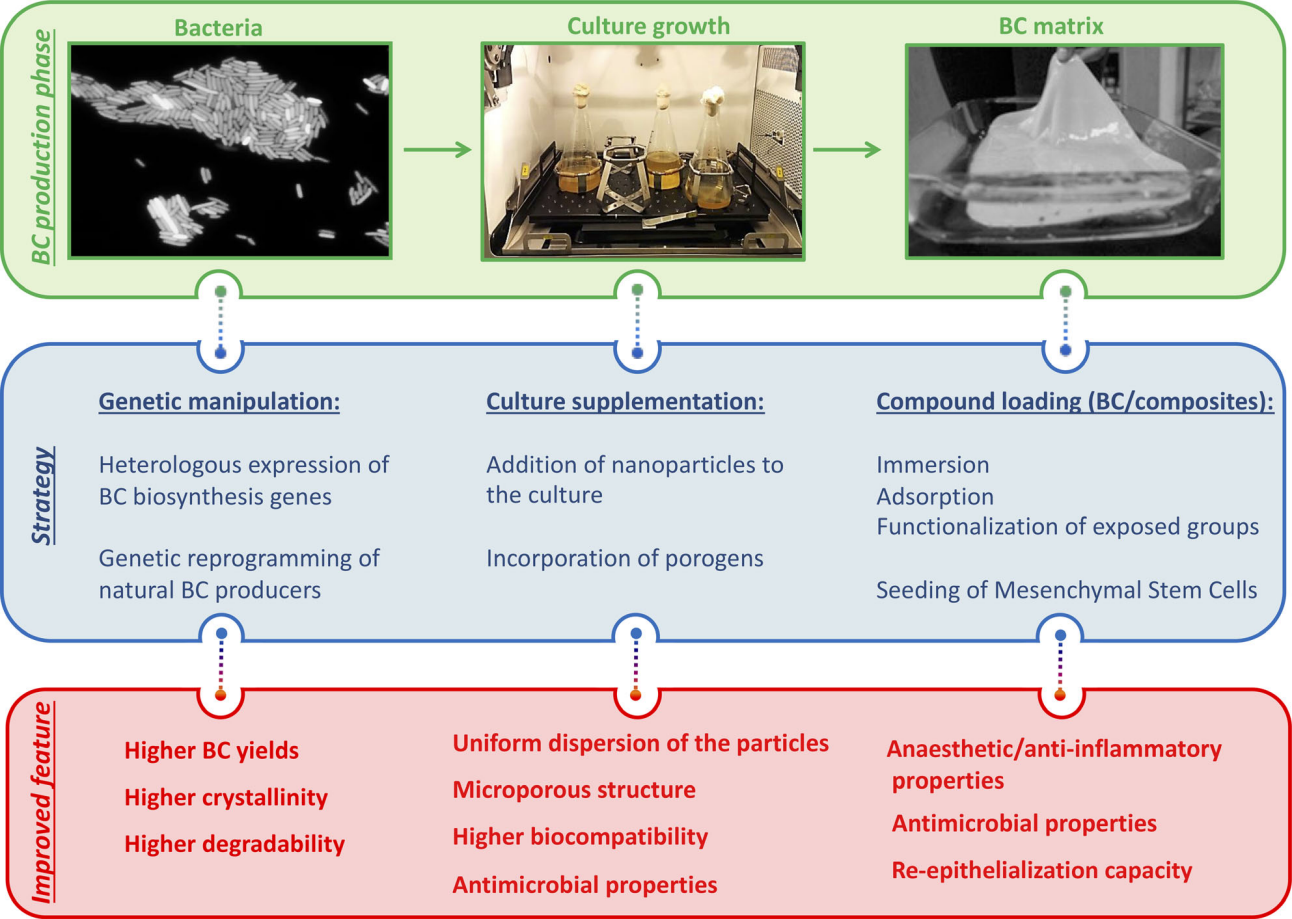
Schematic summary of the processes involved in the bacterial cellulose production (upper panel), the strategies followed for its upgrading (middle panel) and the improvements obtained in each step (lower panel). The BC matrix image was reprinted from Biomaterials, Vol 27 (2), W. Czaja, A. Krystynowicz, S. Bielecki, R.M. Brown Jr., Microbial cellulose – the natural power to heal wounds, Pages No. 145–151, Copyright (2006), with permission from Elsevier.

The points mentioned address the efforts applied to this date towards the optimization of BC as a superior wound dressing agent. Such improvements were performed at all phases of BC production resorting to different strategies for the achievement of a higher performance as summarized in Fig. 6. However, these efforts still do not meet all the challenges that need to be addressed in a near future.

## Marketed bacterial cellulose‐based wound dressing products

Although BC has intrinsic features that encourage its usage as wound dressing, in particular for burn wounds, its commercial dissemination is not exhaustively exploited. Even after all the studies that demonstrate that modifying pure BC in order to enhance its properties for a specific function achieving, for instance, better healing rates or antibacterial properties of the dressing, there are not many commercial products that are BC‐based, for wound dressing applications. The manufacturers are mainly located in the United States of America, Brazil and Poland. In the field of medicinal drug delivery systems, only polyhexamethylene biguanide (PHMB)‐supplemented BC (*Suprasorb X+ PHMB*) commercialized by the *Activa Healthcare, L&R Company*, has been marketed as wound dressing (Wild *et al*., 2012). A company branded *Biofill* offers several products that are BC‐based for wound dressing application such as *Biofill* to be used as temporary skin (substitute for ulcers and burns) allowing pain relief, reduced infection and faster healing; *BioProcess* intended for usage on ulcers and burns with antibacterial properties and increased healing rate; and *Gengiflex* to be used as dental implants or grafting material, favouring the recovery of periodontal tissue, reducing inflammatory response and surgical steps (Picheth *et al*., 2017).

**Figure 7:**
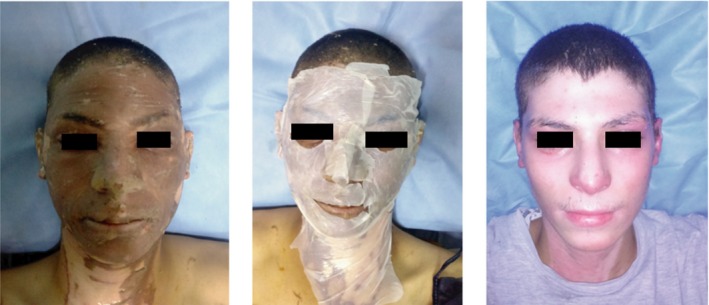
Time evolution of a facial burn wound during 28 days. On the left, before cleaning; on the middle, with BC sheets applied; and on the right, healed wound after the 28 days. Reprinted from Burns Vol 44, Ahmed Aboelnaga, Moustafa Elmasrya, Osama A. Adly, Mohamed A. Elbadawy, Ashraf H. Abbas, Islam Abdelrahman, Omar Salah, Ingrid Steinvall, Microbial cellulose dressing compared with silver sulphadiazine for the treatment of partial thickness burns: A prospective, randomized, clinical trial, Pages No. 1982–1988, Copyright (2018), with permission from Elsevier.

Several other companies present similar products such as *Membracel* commercialized by *Vuelo Pharma* to act as temporary skin substitute for ulcers, burns and lacerations allowing fast skin regeneration; *xCell* commercialized by *Xylos Corporation* to be used as wound dressing for venous ulcer wounds, promoting autolytic debridement, pain relief and accelerating granulation; *Nanoderm* and *Nanoderm Ag* from *Axcelon Biopolymers Corp*. that prevent infections due to their antimicrobial properties; *Nanoskin* produced by *Innovatec* intended to be used as substitute blood vessels; and linfatics, lesions of tegument, facial peeling, infectious dermolysis, abrasion of tattoos and chronic ulcers, allowing the release of gases while obstructing the entry of microorganisms (Czaja *et al*., 2007; Picheth *et al*., 2017). Following the research performed in Lodz University of Technology (Poland), *Bowil Biotech* started the production and commercialization of BC wound dressings using the product name *Celmat*. Recent clinical trials demonstrate the BC wound dressing efficiency in human burn wounds (Fig. 7) where commercial dressings were used on wounds after being cleaned with normal saline and any bullae or debris removed. Microbial cellulose (*EpiProtect* ^®^ from S2Medical AB, Sweden) sheets were applied under aseptic conditions, and in 28 days, the wound was healed (Aboelnaga *et al*., 2018).

## Future perspectives

The potential of BC usage in different industrial fields was recently evaluated revealing the emerging applications of BC‐based technology and anticipating a growing BC market in the order of 15% (Digital Journal, 2018).

BC is presently being used to develop bio‐based, commercial 3D printing materials, as an alternative to chemical products, such as resins, synthetic thickeners, strengtheners and plastics. Another advantage of 3D‐printed BC is the possibility to create adjustable dressings.

3D‐printable bioactivated BC/alginate hydrogel can offer a platform for the development of biomedical devices such as wearable sensors and drug‐releasing materials, allowing to monitor the condition of patients’ wounds while in the hospital (Leppiniemi *et al*., 2017). For example, the incorporation of specific sensors into the cellulosic material that glow with a different intensity in reaction to changes in the wound's pH level can be accessed using a UV lamp without removing the dressing. This procedure allows the healing process to continue unimpeded and can be easily followed at home by the patient. Another approach used silver ink to print the measurement electrodes onto a BC/polyurethane film allowing temperature reading into a computer and, in theory, to access instantly patient's wound condition. This film is assembled with the 3D‐printed wound care gel, with active ingredients of alginate, glycerol and BC, to promote the dress healing treatment. This 3D nanocellulose dress strategy enables the growth of healthy skin cells around a wound, creating a solution where a protein attaches to a 3D‐printed adhesive bandage favouring the cell growth – in this way, the healed area around the wound will stay malleable, instead of growing scab tissue.

The evolution to a personalized medicine approach when treating patients with chronic wounds, such as the case in diabetes, is inevitable, since management of skin tissue impairment is more than just managing a wound. A comprehensive assessment of the individual's health, nutrition, comorbidities and activity levels are key features to formulate an adequate treatment strategy. In this regard, the possibilities to design and to control specific drug release and to monitor the wound healing process in real time, without interference, are a challenging goal and appear to be within reach.

In conclusion, BC may be considered as a potential builder for nano‐based materials such as composites, films, foams and gels presenting distinctive properties. Such materials appear as promising alternatives to petroleum‐based ones, with the advantage of being environmentally friendly and recyclable. BC presents mechanical and physical outstanding properties that emerge from its unique 3D structure. BC is also biodegradable, non‐toxic and biocompatible, and is produced with a unique native purity, which allows for its direct utilization. BC‐based materials have been explored in a diversity of possible applications for biomedical purposes, in particular to develop improved dressing materials for severe wound healing. Novel scientific works, developing BC‐based materials for biomedical applications, have disclosed the potential of these materials, and in spite of further studies on *in vivo* biocompatibility, a promising future for BC materials is already revealed.

## Conflict of interest

None declared.
